# Mechanisms of Hamstring Injury in Professional Soccer Players: Video Analysis and Magnetic Resonance Imaging Findings

**DOI:** 10.1097/JSM.0000000000001109

**Published:** 2022-11-25

**Authors:** Aleksi Jokela, Xavier Valle, Jussi Kosola, Gil Rodas, Lluís Til, Maria Burova, Pavel Pleshkov, Håkan Andersson, Giulio Pasta, Paolo Manetti, Gabriel Lupón, Ricard Pruna, Alvaro García-Romero-Pérez, Lasse Lempainen

**Affiliations:** * Faculty of Medicine, University of Turku, Turku, Finland;; † Department of Orthopaedics and Traumatology, Turku University Hospital, Turku, Finland;; ‡ FC Barcelona, Medical Department, Barcelona, Spain;; § Department de Cirurgia de la Facultat de Medicina, Universitat Autònoma de Barcelona, Barcelona, Spain;; ¶ Department of Physical Activity and Health, Paavo Nurmi Centre, University of Turku, Turku, Finland;; ║ Human Performance Department SL Benfica, Lisbon, Portugal;; ** FC Zenit, Saint Petersburg, Russia;; †† High Performance Center, Växjö, Sweden;; ‡‡ Parma Calcio, Parma, Italy;; §§ Girona FC, Medical Services, Barcelona, Spain;; ¶¶ Watford FC, Injury Prevention and Rehabilitation Department, Watford, United Kingdom;; ‖‖ Physiotherapy Department, Universidad Camilo José Cela, Madrid, Spain; and; *** FinnOrthopaedics/Hospital Pihlajalinna, Turku, Finland and Department of Physical Activity and Health, Paavo Nurmi Centre, University of Turku, Turku, Finland.

**Keywords:** hamstring, muscle injuries, injury mechanism, video analysis, magnetic resonance imaging, single-tendon

## Abstract

To describe the injury mechanisms and magnetic resonance imaging (MRI) findings in acute hamstring injuries of male soccer players using a systematic video analysis.

**Design:**

Descriptive case series study of consecutive acute hamstring injuries from September 2017 to January 2022.

**Setting:**

Two specialized sports medicine hospitals.

**Participants:**

Professional male soccer players aged between 18 and 40 years, referred for injury assessment within 7 days after an acute hamstring injury, with an available video footage of the injury and positive finding on MRI.

**Independent Variables:**

Hamstring injury mechanisms (specific scoring based on standardized models) in relation to hamstring muscle injury MRI findings.

**Main Outcome Measures:**

Hamstring injury mechanism (playing situation, player/opponent behavior, movement, and biomechanical body positions) and MRI injury location.

**Results:**

Fourteen videos of acute hamstring injuries in 13 professional male soccer players were analyzed. Three different injury mechanisms were seen: mixed-type (both sprint-related and stretch-related, 43%), stretch-type (36%), and sprint-type (21%). Most common actions during injury moments were change of direction (29%), kicking (29%), and running (21%). Most injuries occurred at high or very high horizontal speed (71%) and affected isolated proximal biceps femoris (BF) (36%). Most frequent body positions at defined injury moments were neutral trunk (43%), hip flexion 45-90 degrees (57%), and knee flexion <45 degrees (93%). Magnetic resonance imaging findings showed that 79% were isolated single-tendon injuries.

**Conclusions:**

According to a video analysis, most hamstring injuries in soccer occur during high-speed movements. Physicians should suspect proximal and isolated single-tendon—most often BF—hamstring injury, if represented injury mechanisms are seen during game play. In addition to sprinting and stretching, also mixed-type injury mechanisms occur.

## Abstract 2

Supplemental Digital Content is Available in the Text.

## INTRODUCTION

Hamstring injuries are among the most common sports injuries and can cause notable disability in athletes.^1–3^ These injuries occur especially in sports involving stretching, jumping, sudden turns, and high-speed running with rapid acceleration and deceleration.^1,2,4,5^

Two different hamstring injury mechanisms have been previously described in the literature: stretching-type and sprinting-type.^6,7^ The stretching-type hamstring injury has been shown to most commonly affect the proximal tendon of the semimembranosus (SM)^8^ or biceps femoris (BF).^7^ By contrast, the sprinting-type hamstring injury most often affects the long head of the BF (BFlh),^9^ whereas the most severe avulsions of BF, SM, and/or semitendinosus (ST) usually occur as a result of a rapid forceful hip flexion with the ipsilateral knee in extension.^10^

However, in certain cases, the hamstring injury mechanisms are not so easily classifiable, as the injury inciting actions may involve different movements that make hamstrings susceptible to injury. The injuries may combine biomechanical characteristics from both sprinting and stretching-type mechanisms. Worth^11^ described that the most common hamstring injury situation in Australian football is when the player tries to pick up a ball from the ground while running at full speed, including both sprinting and trunk flexion causing full stretch and eccentric contraction of the hamstrings. Therefore, in our opinion also, “mixed-type” injury mechanisms can occur in hamstring injuries.

Hamstring injuries have been widely investigated in the literature, but little is known about the specific injury mechanisms.^7,12^ In addition, the descriptions of the injury mechanisms have been traditionally categorized to sprinting and stretching, and the injuries were not observed by the researchers in most of the studies.^6,8,9^ The value of video analysis to understand injury mechanisms and injury patterns has been previously demonstrated in sports.^13–17^ The knowledge of the different hamstring injury mechanisms can help in identifying the injury location and the severity of injury.^6,8–10^ Video analysis of hamstring injuries can be very helpful not only in patient consultations but also in designing the best rehabilitation and prevention programs, as well as rendering an accurate prognosis.^18^

From our review of the literature, there is a paucity of information on the precise mechanisms of hamstring injuries in soccer players. We hypothesize that video analysis could be helpful in understanding the pattern of hamstring injuries in soccer players. The aim of this study was to describe the mechanisms, situational patterns, biomechanics, and magnetic resonance imaging (MRI) findings related to hamstring injuries in professional male soccer players using a systematic video analysis.

## METHODS

### Subjects

Professional male soccer players with an acute hamstring injury were included from 2 private departments at specialized sports medicine hospitals in Finland and Spain from September 2017 to January 2022. The inclusion criterion was as follows: professional male soccer players (aged 18-40 years) with initial and acute onset hamstring pain occurred while training or competing. In addition, subjects had to have a confirmed hamstring injury on an MRI that was performed within 7 days of the date of injury as well as video footage of the injury when it occurred. Exclusion criteria were as follows: pain of nonmusculotendinous cause, recurrent injury without video footage of initial injury, inadequate quality of video footage, or refusal to allow the use of video footage. Narrative descriptions of each case are included in the **Supplemental Digital Content 1** (see **Supplementary file 1**, http://links.lww.com/JSM/A349).

### Video Acquisition and Processing

All included hamstring injuries were broadcasted on television during game play or recorded by a training crew while filming training or playing performance according to the standard protocol. Thirteen injuries occurred during game play, one in game play during training session. All video footage was obtained through the teams' archiving system or through public sources. The videos were stored as a MP4 format in standard quality. Injury sequences were edited using Wondershare Filmora9 V.9.5.3 and iMovie V.10.1.2 software and converted to QuickTime (.mov), allowing frame-by-frame navigation using QuickTime player V.10.4. The faces of subjects and names on player jerseys were blurred to anonymize all the videos. In further video processing, we followed the steps presented in a study published by Serner et al.^17^ To reach a good demonstration of the mechanism of injury, the video was cut from the start of performance before the injury to the stop in performance immediately after injury. In addition, shorter clips were made, which included footage of the specific injury mechanism from each available camera view. This means there was 1 clip of the full situation, as well as one to 4 additional slow motioned clips depending on the number of available camera angles, allowing easy frame-by-frame navigation.

Nine videos were acquired through the teams' own archiving system. One video was acquired through Sky Italia archives, and the use of video footage was authorized by the company. One injury was acquired from personal video footage, filmed by the athlete's training crew. Three videos were accessed by publicly available Internet sources. Three injuries were captured from 1 camera view, 1 from 2 views, 9 from 3 views, and 1 from 4 views. Eight videos were in resolution 1920 × 1080, 5 in 1280 × 720, and 1 in 464 × 848.

### Determination of Injury Movement

Each injury was reviewed and discussed with each injured athlete to determine the specific movement and body position in which the player recalled feeling the pain. This review was performed within 24 to 48 hours of the initial injury in most cases. In cases where the athlete could not determine the specific body position or movement, the assumed time of injury was estimated by 4 authors based on the injury mechanism, body positions, and athlete reactions. Based on this information, the assumed exact injury frame was defined.

### Video Analysis

Athletes' own narrative descriptions were reported focusing on inciting events, the actual moment of pain sensation, and capability to continue playing/training/performing. Subsequently, 4 analysts (2 orthopedic surgeons, a sports medicine physician, and a clinician) independently assessed all videos in real time, slow motion, and frame-by-frame to describe the specific injury mechanisms of the hamstring injuries. The analysts watched the injury tapes on their own computers using a video player that allowed them to view the sequences as many times as needed. Three analysts (an orthopedic surgeon, a sports medicine physician, and a clinician) created specific descriptions of all injury situations focusing on dynamics, biomechanics, and injury mechanisms relevant to hamstring muscles.

Based on a comprehensive model for injury causation^19^ and standardized scoring forms,^13–17^ a specific hamstring questionnaire (see **Supplementary file 2**, **Supplemental Digital Content 2**, http://links.lww.com/JSM/A350) was developed to describe accurately the injury mechanism and the events leading up to the injury. Two analysts (A.J. and X.V.) were asked to fill the standardized form involving specific questions about playing situation, player/opponent behavior, movement, and biomechanical body positions at defined injury moments (see **Supplementary file 2**, **Supplemental Digital Content 2**, http://links.lww.com/JSM/A350). The analysts classified the injury mechanisms independently. Any discrepancies in the analysis were noted and discussed in a consensus meeting where videos were critically viewed again until the consensus was reached. Analysis was performed using Excel 2018 (Version 16.16.27). The cases were further categorized into sprint-related, stretch-related, or mixed-type patterns (including patterns from both sprint-related and stretch-related categorizations) based on the categorization options used by Gronwald et al.^7^

### Magnetic Resonance Imaging Analysis

Four analysts (a radiologist, 2 consultant orthopedic surgeons, and a sports medicine physician) with special expertise and experience in analyzing hamstring MRIs in their daily work independently assessed all magnetic resonance (MR) images to evaluate possible lesions of the hamstring muscles. To be included, axial, sagittal, and coronal fat-suppressed T1-weighted and T2-weighted MRI investigation of the pelvis and both thighs had to be performed within a week after an acute hamstring injury. For interpreting the MRI findings, a consensus was established, with consensus defined as all analysts agreeing, based on the general muscle lesion patterns previously described in the literature.^20–23^ A hamstring muscle was considered injured if it contained high signal intensity compared with the uninjured posterior thigh. A tendon tissue was considered injured if it was thickened and/or had an intratendinous high signal and/or a collar of high signal intensity around it, as compared with the uninjured side. The anatomical location of the injury with involved muscle(s) and tendon(s) was analyzed.

### Ethical Considerations

The study protocol was approved by the Ethics Committee of the Hospital District of Southwest Finland (ETMK 54/1801/2020). All included subjects were informed about the methodology, participated on a voluntary basis, and consent was acquired from all subjects at inclusion according to the Declaration of Helsinki.

## RESULTS

### Subjects

Fourteen videos of acute hamstring injuries of 13 professional male soccer players met the inclusion criteria. One subject sustained a hamstring injury in each leg over a 4-year period of the study. Therefore, 14 cases were included (median age: 23 years, range 20-37). Of the 13 subjects, 1 was a goalkeeper, 4 defenders, 3 midfielders, and 5 forwards. Characteristics of each case are presented in Table 1.

**TABLE 1.** Characteristics of Each Case With Case Number, Age, Position, Injury Location in MRI, Action During Injury, Injury Mechanism, and Joint Angles at Assumed Injury Frame (Trunk, Hip, and Knee)

Video Analysis								
Case	Age	Position	MRI finding	Action During Injury	Injury Mechanism	Trunk	Hip	Knee
1	20	M	Proximal BF	Running (acceleration)	Mixed-type	Flexion 45-90 degrees	Flexion 45-90 degrees	Flexion 45-90 degrees
2	21	F	Proximal BF	Running (in speed)	Sprint	Neutral	Flexion 45-90 degrees	Flexion <45 degrees
3	29	D	Proximal BF	Running (in speed)	Sprint	Flexion <45 degrees	Flexion 45-90 degrees	Flexion <45 degrees
4	21	F	Proximal BF	Change of direction	Sprint	Neutral	Flexion 45-90 degrees	Flexion <45 degrees
5	22	D	Mid-thigh SM	Change of direction	Mixed-type	Neutral	Flexion 45-90 degrees	Flexion <45 degrees
6	24	F	Distal BF	Change of direction	Mixed-type	Flexion <45 degrees	Flexion <45 degrees	Flexion <45 degrees
7	31	M	Proximal BF + ST	Change of direction	Mixed-type	Flexion >90 degrees	Flexion <45 degrees	Flexion <45 degrees
8	24	M	Distal SM	Kicking (with an uninjured leg)	Stretch	Flexion <45 degrees	Flexion 45-90 degrees	Flexion <45 degrees
9	20	F	Proximal BF	Kicking (with an injured leg)	Mixed-type	Neutral	Flexion <45 degrees	Flexion <45 degrees
10	37	D	Distal ST	Kicking (approach to kick)	Mixed-type	Neutral	Flexion <45 degrees	Flexion <45 degrees
11	21	D	Distal BF	Kicking (with an injured leg)	Stretch	Neutral	Flexion >90 degrees	Flexion <45 degrees
12	28	F	Proximal BF + ST	Jumping (landing)	Stretch	Flexion <45 degrees	Flexion >90 degrees	Flexion <45 degrees
13	28	GK	Proximal SM	Reaching for ball	Stretch	Flexion 45-90 degrees	Flexion 45-90 degrees	Flexion <45 degrees
14	20	F	Proximal BF + ST	Shielding	Stretch	Flexion >90 degrees	Flexion 45-90 degrees	Flexion <45 degrees

D, defender; F, forward; GK, goalkeeper; M, midfielder

### Injury Mechanisms

Three different injury mechanisms were seen in video analysis: mixed-type (both sprint-related and stretch-related, 43%), stretch-type (36%), and sprint-type (21%). Video examples of all 3 mechanisms are presented in **Supplemental Digital Content 3 to 6** (see **Supplementary files 3-6**, http://links.lww.com/JSM/A351, http://links.lww.com/JSM/A352, http://links.lww.com/JSM/A353, http://links.lww.com/JSM/A354). All players were able to determine the situation and movement causing the pain in the hamstring area. Characteristics of each case and descriptive information on injury situations are presented in Tables 1 and 2.

**TABLE 2.** Descriptive Information on Injury Situations Categorized Into Different Injury Mechanisms

Descriptive Information on Injury Situations					
	Horizontal Speed	Vertical Speed	Balance	Contact	Open/Closed Chain
Case			*Running*		
1	Moderate	Low	Out of balance	Yes (indirect, before the injury)	Closed chain
2	Very high	Low	In balance	No	Open chain
3	Very high	Low	In balance	No	Open chain
			*Change of direction*		
4	Very high	Low	In balance	Yes (indirect, before the injury)	Open chain
5	Very high	Low	In balance	Yes (indirect, before the injury)	Open chain
6	Very high	Low	In balance	No	Closed chain
7	High	Low	Out of balance	No	Closed chain
			*Kicking*		
8	High	Low	Out of balance	No	Closed chain
9	High	Low	In balance	No	Open chain
10	Very high	Low	Out of balance	No	Closed chain
11	Moderate	Low	In balance	Yes (indirect, before the injury)	Open chain
			*Other*		
12 (jumping)	Zero	High	Out of balance	Yes (indirect, before the injury)	Open chain
13 (reaching for ball)	High	High	Out of balance	Yes (indirect, before the injury)	Closed chain
14 (shielding)	Low	High	Out of balance	Yes (at the time of injury)	Closed chain
			*Most frequent variables*		
Total	Very high 43%	Low 79%	In balance/out of balance 50%/50%	No 50%	Open/closed chain 50%/50% (5/10)

### Magnetic Resonance Imaging Findings

**Figure 1.**
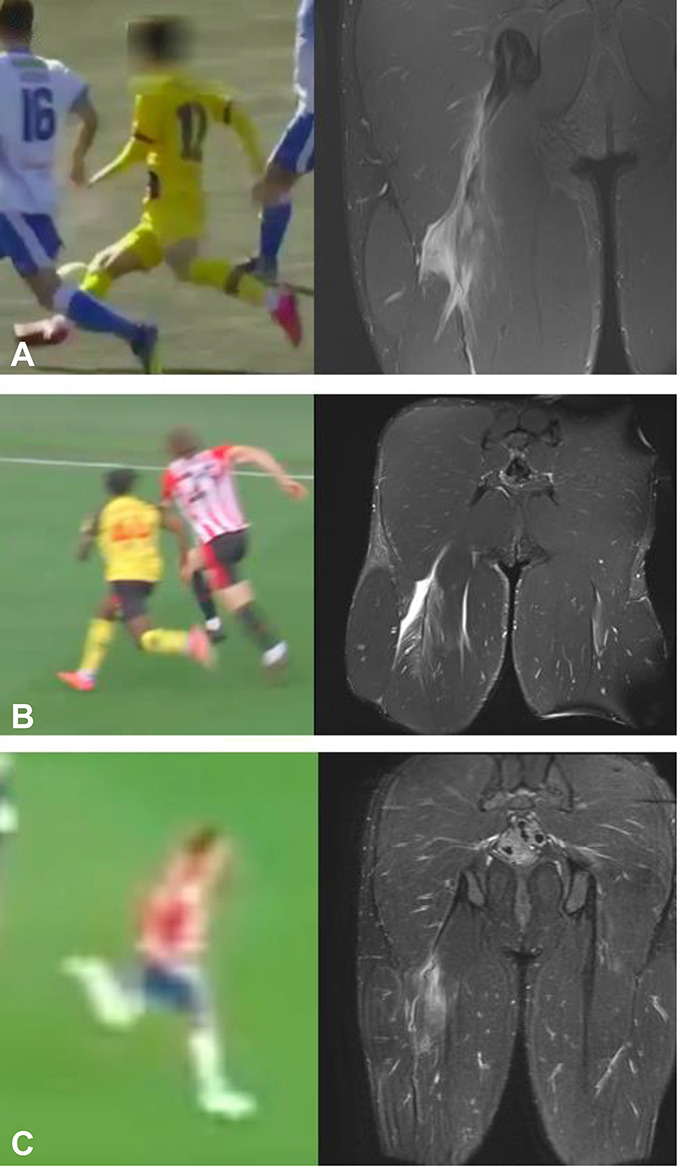
Assumed injury frames and MRI findings in sprint-type injuries. A, Case 4, proximal BF injury. B, Case 2, proximal BF injury. C, Case 3, proximal BF injury.

**Figure 2.**
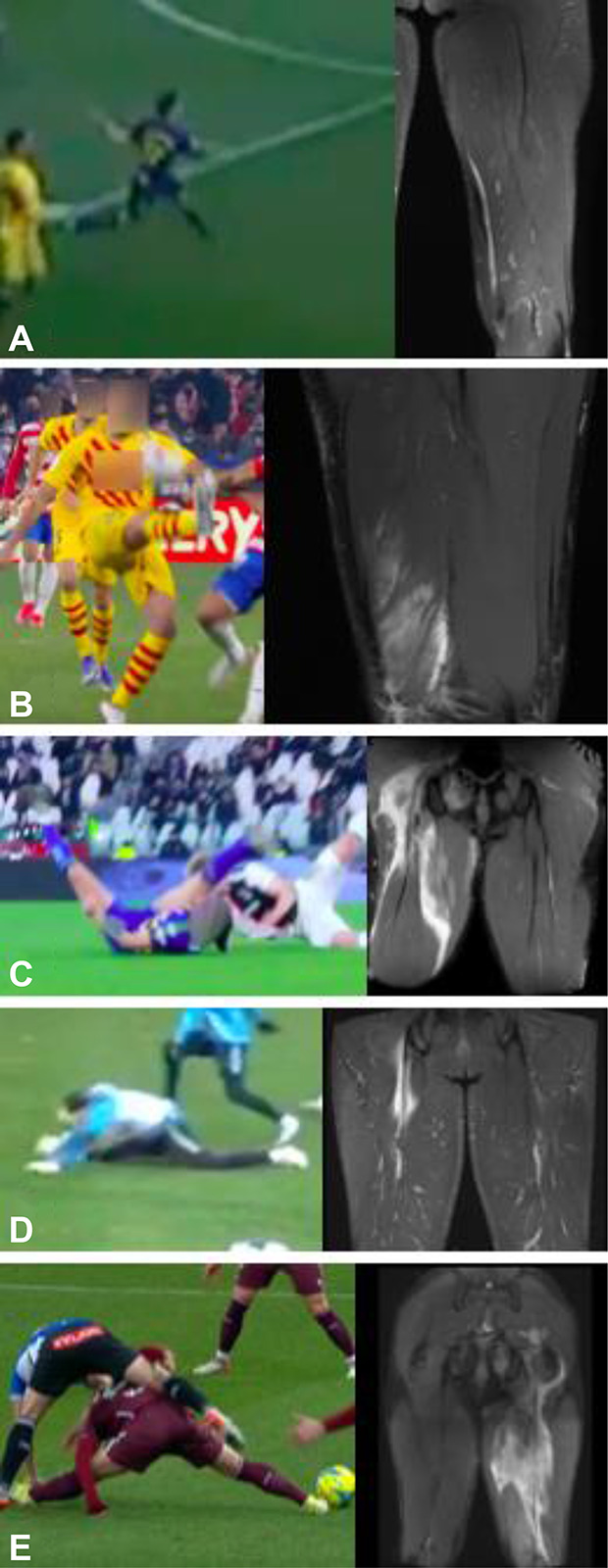
Assumed injury frames and MRI findings in stretch-type injuries. A, Case 8, distal SM injury. B, Case 11, distal BF injury of the MTJ. C, Case 12, proximal BF + ST avulsion. D, Case 13, proximal SM injury. E, Case 14, proximal BF + ST avulsion. MTJ, myotendinous junction

**Figure 3.**
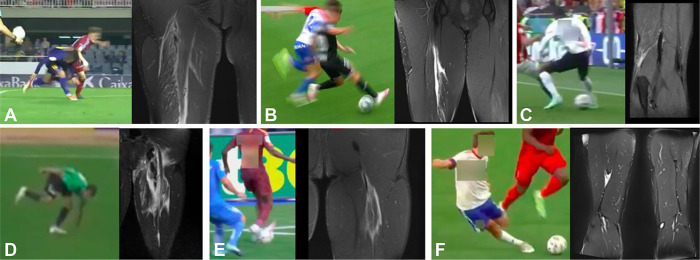
Assumed injury frames and MRI findings in mixed-type injuries. A, Case 1, proximal BF injury. B, Case 5, mid-thigh SM injury. C, Case 6, distal BF avulsion. D, Case 7, proximal BF + ST avulsion. E, Case 9, proximal BF injury. F, Case 10, distal ST injury of the MTJ. MTJ, myotendinous junction

Magnetic resonance imaging findings with corresponding injury mechanisms are presented in Table 1. Of the 14 injures, 10 involved BF (71%); 5 cases were isolated proximal (36%) and 2 distal BF injuries (14%), and 3 injuries were avulsions involving proximal BF and ST tendons (21%). Three cases were isolated SM injuries (21%), of which 1 was proximal, 1 distal, and 1 was located in mid-thigh. One injury affected the distal myotendinous junction of the ST (7%). MR images with corresponding assumed injury frames are presented in Figure 1 (sprint-type), Figure 2 (stretch-type), and Figure 3 (mixed-type).

## DISCUSSION

This study shows that video analysis is a helpful tool in evaluating hamstring injury mechanisms in soccer. We found that soccer players had mainly single-tendon hamstring injuries, but also, more severe avulsions are possible. In addition to sprinting and stretching, we described the mixed-type injury mechanism, which included typical patterns from both of these mechanisms. Most cases (93%) involved knee flexion <45 degrees, and in 6 of the cases, we found the fully extended knee. Hip and trunk flexion were also typical biomechanical factors associated with hamstring injury.

### Implications to Clinical Diagnosis of Hamstring Injuries in Soccer

Clinical diagnosis of hamstring injury is based on suspicion after acute onset symptoms during exercise combined with one of the described injury mechanisms. The clinical diagnosis needs to be confirmed with imaging tests (MRI and/or ultrasound).^24^ In addition, video analysis allows clinicians to deeply understand the mechanisms and factors having influence on the injury process, which can be helpful in diagnosis, treatment, and even in prevention of hamstring injuries. In soccer, which requires rapid acceleration, deceleration, and sudden turns, hamstring injuries are common, especially in the BFlh.^2,25^ We found all 3 of the sprint-type injuries affecting proximal BF, 2 occurred during linear high-speed running and 1 during curved high-speed running (change of direction). Two sprint-type injuries involved neutral trunk, and in 1 case, there was <45 degrees trunk flexion. All sprint-type cases included hip flexion angle of 45-90 degrees and knee flexion <45 degrees at an assumed injury frame, which is a typical position to the late swing phase during running.^26^ Based on our and other groups' previous findings, we recommend to suspect a proximal BF injury in the case of acute posterior thigh pain that occurred during linear or curving high-speed running.^6,7^

The stretch-type injuries usually occur during a side or sagittal split movement, high kick, or stretching.^27^ In our sample, of the 5 stretch-type injuries (36% of all injuries), 2 affected SM, 2 were proximal avulsions (BF + ST), and 1 affected distal BF. All of the stretch-related injuries occurred during a rapid change of movement involving hip flexion and knee extension. Gronwald et al^7^ found similar results to our findings, as all of the stretch-related cases happened during a change of movement with the knee going from flexion to extension with a knee angle of <45 degrees at an assumed injury frame. The stretching-type of proximal hamstring injuries has been described in several disciplines, such as dance, tennis, soccer, and aerobics,^27^ and even causing proximal avulsions in violent overstretching movements in waterskiing, judo, soccer, rugby, and bull riding.^28–31^ Proximal SM has been found to be the most common injury location during stretch-type injury.^6,8,27^

The 6 mixed-type injuries involved high-speed running or acceleration combined with stretch-related movement (lunging, landing, or kicking), and the injury locations varied widely: 2 proximal BFs, proximal 2 tendon avulsions (BF + ST), mid-thigh SM, distal BF, and distal ST. Rapid movements involving both sprinting and stretching can be very difficult to identify based on only real-time eye witness or athlete's own recollection. If the injury moment is captured on video, the video analysis with slow motion and video stoppage enables specific and detailed assessment of injury mechanism. We used the frame-by-frame approach to detect the exact biomechanics during these high-speed playing situations. In our Case 1, the player started to accelerate while the opponent pushed his shoulder, forcing him to a trunk flexion position and elongating hamstring muscles while running, which caused proximal BF injury. Our Case 1 is identical to the most common hamstring injury situation in Australian football described by Worth.^11^ The mixed-type injury mechanism is very important to be recognized and incorporated to biomechanics studies, as the prevalence is high and they do not follow the typical patterns of sprint or stretch-type injuries. Clinicians should be aware of the importance of examination and imaging because the location and severity of the injury are very difficult to predict after this mechanism.

Gronwald et al^7^ found that both stretch-related and sprint-related injury mechanisms affected BF most often (70% and 88%). Stretching-type hamstring injury has been classically defined as excessive hip flexion with the ipsilateral knee extended.^8,27^ Instead, Gronwald et al^7^ defined stretch-related patterns as kicking (open chain) and breaking or stopping (closed chain). The differences in categorization may also explain the difference in the prevalent injury type (SM vs BF), as the classical categorization involves typically sagittal split movement in which SM is dominantly elongated,^8^ whereas the braking movements may involve high speeds during running before stopping the movement, explaining the high prevalence of BF injuries.^7^ As Gronwald et al^7^ categorized each injury into either stretching or sprinting, some of their stretch-related injuries would have met our criteria of the mixed-type pattern. This may also explain the high prevalence of BF injuries among stretch-related injuries, as BF has been found to be the typical injury type in sprint-type injuries.^9^

### Implications to Clinical Treatment and Rehabilitation of Hamstring Injuries in Soccer

Most of the hamstring injuries are mild strains that heal well conservatively, but some severe ruptures may require surgical treatment, especially in professional athletes.^10^ Soccer players often return to play after 4 to 7 weeks of rehabilitation after an acute hamstring injury.^6^ The differences in time to return can be found between different injury mechanisms and rehabilitation protocols.^6^ Video analysis can be helpful in quick evaluation after the injury. Immediate remote consultation to an expert can be performed by the medical department of the player's team, as the video footage and the imaging tests allow to consensuate the correct treatment, conservative or surgical. For rehabilitation, video analysis of the initial injury mechanism can be used to simulate injury-prone situations to test whether the player is ready to return to play or not. Therefore, we highly recommend video analysis to be used among sports medicine clinicians when designing rehabilitation and prevention programs of hamstring injuries.

### Implications to Clinical Prevention Strategies of Hamstring Injuries in Soccer

Our results suggest that sport-specific exercises including movements with eccentric loads on hamstrings, high-speed running, and combinations of rapid movements, focusing on trunk control, may be useful in the prevention of hamstring injuries. Since BF is the most commonly injured hamstring muscle in soccer,^7^ that should probably be taken into consideration in prevention programs. Injury to proximal BF is common during sprinting.^6,9^ During high-speed running, it seems that the highest risk of hamstring injury is in the late part of the swing phase, right before the foot strikes the ground.^26^ In addition to the late swing, the early stance phase seems to be a critical point at which hamstring injury is more likely to occur.^32^ Green et al^33^ described that fatiguing during high-speed running can lead to changes in muscle strength and flexibility qualities. Several risk factors for hamstring injury have been found, including older age, previous injuries, and poor trunk and hamstring muscle control.^33^ Therefore, special attention is recommended to be paid to prevention programs for athletes, who are older or have had previous injuries. Video analysis is helpful in analyzing injury situations or specific biomechanics during playing soccer. This can reveal risky patterns, such as poor trunk control or running technique, which can be taken into consideration when developing individual and demand-specific prevention programs. These programs should be implemented to realistic in-field situations simulating real-life injury mechanisms, which can be created with the help of video analysis of hamstring injuries.

### Strength and Limitations

Our study's strength is that it offers clinicians a novel injury assessment tool that is easily accessible and can quickly evaluate the need of further measures in cases of acute hamstring injury in soccer. The hamstring injuries in this study were also confirmed using MRI, which is a gold standard in diagnostics and helpful guide in decision-making. We had the information of the pain evaluation in all cases, which makes the assumed injury frames more reliable. The limitations include biomechanical analysis of body positions, which seems to be demanding, and therefore, we used only rough estimations (45 degrees range) in our visual analysis of joint angles. Despite the relatively small sample size, we showed the value of video analysis in more accurate assessment of injury mechanisms. With the combination of athlete's recollection, video analysis, and MRI findings, we gained a decent description of mechanisms, types, and patterns of injury. Including mixed-type injury mechanism in our analysis offered novel information on injury patterns. Further research using video footage with bigger sample sizes, investigating the correlation between injury mechanisms and different types of injury with grading, is suggested. In addition, as there are many different mechanisms of hamstring injury, further analysis should be performed to get more detailed information of the forces related to each anatomical site of the posterior thigh.

## CONCLUSIONS

In summary, video analysis is a helpful tool for a physician in understanding injury mechanisms and their relation to types of hamstring injury. Using video analysis, a clinician can evaluate the most likely diagnosis immediately after injury, which offers essential information about the nature and prognosis of injury. In addition, video analysis can help to develop more specific preventive programs. Hamstring injury mechanisms typically involve hip flexion, knee extension, and trunk flexion. Single-tendon hamstring injuries (mostly BF) are typical in soccer and mainly occur due to high-speed movements involving high eccentric load of the hamstring muscles. Mixed-type injury mechanisms also occur, which include patterns from both sprinting-type and stretching-type injury mechanisms.
